# Association between thyroid hormone levels and insulin resistance and body mass index

**DOI:** 10.12669/pjms.316.7560

**Published:** 2015

**Authors:** Neval Aksoy, Mustafa Taner Yeler, Nilhan Nurlu Ayan, Ali Ozkeskin, Zeynep Ozkan, N.Ozden Serin

**Affiliations:** 1 Neval Aksoy, Gaziosmanpasa Taksim Education and Research Hospital, Department of Biochemistry, Elazig-Turkey; 2 Mustafa Taner Yeler, Gaziosmanpasa Taksim Education and Research Hospital, Department of Biochemistry, Elazig-Turkey; 3 Nilhan Nurlu Ayan, Gaziosmanpasa Taksim Education and Research Hospital, Department of Biochemistry, Elazig-Turkey; 4 Ali Ozkeskin, Gaziosmanpasa Taksim Education and Research Hospital, Department of Internal Medicine, Elazig-Turkey; 5 Zeynep Ozkan, Elazig Education and Research Hospital, Department of General Surgery, Elazig-Turkey; 6 N.Ozden Serin, Gaziosmanpasa Taksim Education and Research Hospital, Department of Biochemistry, Elazig-Turkey

**Keywords:** Body mass index, Insulin resistance, Thyroid stimulating hormone

## Abstract

**Objective:**

Previous studies have shown an association between thyroid function and insulin resistance and obesity. We compared insulin resistance and body mass index (BMI) in patients with normal TSH levels (2.5–4.2 µIU/mL), patients diagnosed with subclinical hypothyroidism, and healthy control subjects.

**Methods:**

The study included 104 subjects and was conducted at the Taksim Education and Research Hospital. The subjects were divided into three groups according to TSH levels: Group 1 (high-normal), TSH levels were 2.5–4.2 µIU/mL (n=33); Group 2 (subclinical hypothyroidism), TSH levels were 4.2–10 µIU/mL (n=42); and Group 3 (healthy control), TSH levels were 0.27–2.5 µIU/mL (n=29). The fT3 and fT4 levels were within normal limits in all groups. Insulin resistance and BMI were compared among groups. The homeostasis model assessment of insulin resistance (HOMA-IR) was used to estimate insulin resistance.

**Results:**

HOMA-IR and BMI were not significantly different among groups (p>0.05). A significant positive correlation was found between BMI and HOMA-IR in the high-normal TSH (p>0.059) and subclinical hypothyroidism (p>0.05) groups.

**Conclusions:**

HOMA-IR and BMI are important for the assessment of diabetes and cardiovascular diseases. We found no significant difference in HOMA-IR and BMI values among the three TSH reference range groups.

## INTRODUCTION

Hypothyroidism is a clinical syndrome caused by reduced thyroid hormone secretion from the thyroid gland. Subclinical hypothyroidism is diagnosed when thyroid hormone levels are within the normal reference range (0.45–4.5 mIU/L), but thyroid stimulating hormone (TSH) is elevated and overt thyroid disease is diagnosed when the serum thyroid hormone levels (free T4, with or without T3) are abnormal. Subclinical hypothyroidism is classified as mildly elevated TSH (4.5–10 mIU/L) or markedly elevated TSH (≥10 mIU/L) with normal fT4 levels in both categories.^1^

Hyporthyroidism is more common in females than males and its prevalence increases with age.^2^ Subclinical hypothyroidism is the most common thyroid dysfunction and is usually asymptomatic; however, findings suggestive of hypothyroidism may be seen in 30% of patients.^3^,^4^ Some studies suggested that the upper normal TSH limit can be reduced which level about 2.0-2.5 mU/L.^5^,^6^

In their investigation of the association of TSH levels with vascular disease and high cholesterol, Volzke et al.^7^ found that patients with TSH levels between 2.5 and 4 mU/L were at increased risk of cardiovascular disease. Recent studies have shown that increased serum TSH levels are associated with cardiovascular disease, psychiatric and mental disorders, and the development of overt thyroid dysfunction.^8^ The clinical spectrum of hyporthyroidism varies according to the severity of thyroid dysfunction.^1^

Peppa M et al. reported that endocrine disorders (polycysticovarysyndrome (PCOS), adrenal disordersandthyroidfunctionabnormalities) has been associated with glucose and insulin metabolism disorders.^9^ Several studies have found an association of thyroid function with body mass index (BMI) and insulin resistance based on the homeostasis model assessment of insulin resistance (HOMA-IR), a widely used index of insulin resistance.^10^-^13^

We compared HOMA-IR and BMI values in patients with high-normal TSH levels, those with subclinical hypothyroidism, and in euthyroid healthy control subjects.

## METHODS

We recruited 104 (40 males, 64 females) subjects aged 18–60 years from the Internal Medicine Clinic of Taksim Research and Training Hospital, a tertiary-care hospital in 2012. Patients diagnosed with subclinical hypothyroidism as a result of a clinical condition or surgery and receiving levothyroxine were included in the study. Patients diagnosed with hyperthyroidism and receiving antithyroid treatment, or those diagnosed with diabetes mellitus (DM), chronic renal or liver disease, congestive heart failure or any other systemic illness, or receiving any hormone treatment were excluded from the study. Our study was approved by the local ethics committee.

Venous blood samples were taken from all participants to measure biochemical parameters. The thyroid function profile (TSH, fT4 and fT3), fasting glucose, and fasting insulin were measured after an overnight fast. BMI was measured in all participants.

HOMA-IR was used to estimate insulin resistance according to the formula:

Fasting glucose (mg/dL) x fasting insulin (µU/mL)/405.^14^

BMI was calculated according to the formula: weight (kg)/height (m^2^).^15^

**Fig.1:**
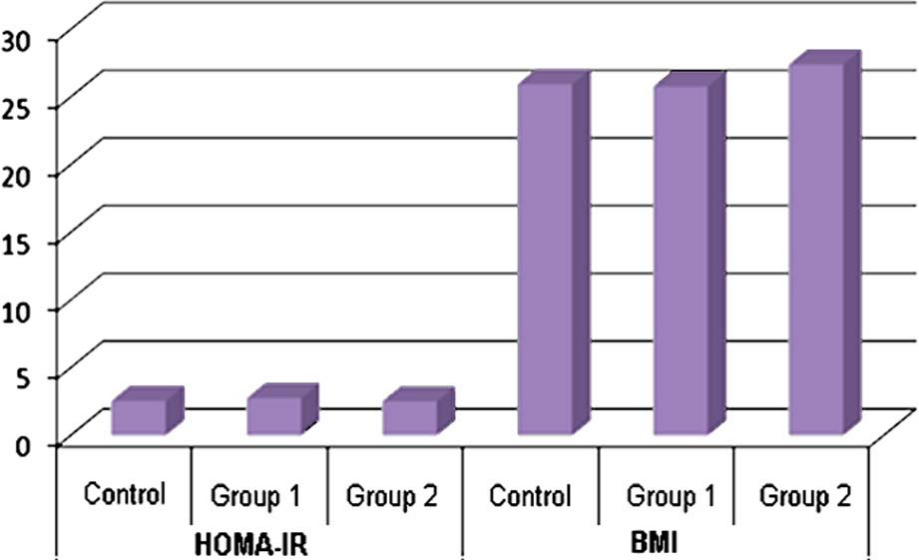
The bar-charts graphics of HOMA-IR & BMI in groups.

Serum glucose, insulin, TSH, fT3, and fT4 levels were assayed using Roche Diagnostics kits and a Roche modular PP-EE chemistry analyzer (Roche Diagnostics GmbH, Mannheim, Germany).

The 104 subjects included in the study were divided into three groups according to TSH levels: Group 1 (high-normal), TSH levels were 2.5–4.2 µIU/mL (n=33); Group 2 (subclinical hypothyroidism), TSH levels were 4.2–10 µIU/mL (n=42); and Group 3 (healthy control), TSH levels were 0.27–2.5 µIU/mL (n=29). The fT3 and fT4 levels were within normal limits in all groups.

The statistical tests were conducted using the Statistical Package for the Social Sciences for Windows 17.0 (SPSS Inc, Chicago, IL, USA). The results are expressed as means ± standard deviation. The Kruskal–Wallis test was used to make among-group comparisons of qualitative data. The association between BMI and HOMA-IR was assessed using Spearman’s correlation analysis.

## RESULTS

The study included 40 male and 64 female patients. We found no statistically significant differences in age, sex, or demographic characteristics among groups. The mean age of participants was 43 years old.

The mean HOMA-IR and BMI values were not significantly different among groups (p>0.05; Table-I). Furthermore, we found no significant difference between the groups with regard to mean HOMA-IR and BMI (p>0.05; Table-I).

**Table-I:** HOMA-IR and BMI in all groups.

	Groups	n	X	SD	KW	p
HOMA-IR	Control	29	2,527	1,436	0,262	0,877
	Group 1	33	2,735	1,814		
	Group 2	42	2,506	1,663		
BMI	Control	29	25,932	5,082	2,109	0,348
	Group 1	33	25,748	5,976		
	Group 2	42	27,371	5,092		

*HOMA-IR:* Homeostasis Model Assessment of Insulin Resistance. *BMI:* Body Mass Index.

BMI and HOMA-IR were not significantly correlated in the control group.(r=0.175; p>0.05; Table-II). However, the correlation analysis revealed significant positive associations between BMI and HOMA-IR in Group 1 accounting for 71.1% of the variance (r=0.711; p<0.05), and in Group 2 accounting for 42.6% of the variance (r=0.426; p<0.05). These findings indicate that as HOMA-IR increased, BMI increased.

**Table-II:** Association of BMI and HOMA-IR in the groups.

			n	r	p
Control	BMI	HOMA-IR	29	0,175	0,363
Group 1	BMI	HOMA-IR	33	0,711	0,000
Group 2	BMI	HOMA-IR	42	0,426	0,005

## DISCUSSION

Hypothyroidism has been associated with glucose and insulin metabolism disorders that affect insulin secretion in response to glucose, hyperinsulinemia, altered peripheral glucose disposal, and insulin resistance.^7^

DM and thyroid function disorders are the two most common endocrine disorders worldwide.^16^ The first study to show a relationship between DM and thyroid disorders was published in 1979 and several subsequent studies have supported those findings.^16^ The prevalence of thyroid disorders in patients with DM varies across countries owing to differences in diagnostic criteria, iodine intake, TSH assay sensitivity, and population diversity.

The relationship between thyroid disorders and DM is characterized by a complex interdependent interaction. Screening for thyroid disorders, particularly subclinical dysfunction, is warranted in patients with DM because most patients are asymptomatic.^16^

Several studies have shown an association between thyroid function and insulin resistance. Maratou et al.^10^ found that fasting and postprandial plasma insulin levels and the HOMA index were higher in patients with hypothyroidism and subclinical hypothyroidism than in euthyroid individuals; however, plasma glucose levels were not significantly different among groups.

Sapna et al.^17^ reported that insulin and HOMA-IR levels were significantly higher in patients with subclinical hypothyroidism than in euthyroid subjects. Additionally, mean TSH levels were moderately positively correlated with insulin and HOMA-IR.

Singh at al.^11^ found a significant positive correlation between TSH levels and HOMA-IR in hypothyroid patients. Thyroid dysfunction causes changes in carbohydrate and lipid metabolism, which are risk factors for cardiovascular disease.

Garduño-Garcia J et al.^18^ and Sridevi et al.^19^ found that fasting insulin and HOMA-IR were associated with serum thyroid hormone, but not TSH levels. Al Sayed et al.^12^ found that insulin levels were significantly higher in Kuwaiti females with subclinical hypothyroidism than in the control group; however, HOMA-IR levels were not significantly different between the groups. Our finding that HOMA-IR did not differ among groups is consistent with that of Al Sayed et al.^12^

Lambadiari et al.^20^ found that HOMA-IR was positively correlated with fT4 and fT3 levels in patients with type 2 DM. Moreover, the authors found a positive correlation between normal-high thyroid hormone levels and insulin resistance, and speculated that thyroid hormones may be involved in the pathological process underlying the development of type 2 DM. In contrast, we did not find a significant difference in HOMA-IR and BMI values between patients with high-normal TSH and healthy controls with normal TSH levels.

Ren et al.^13^ investigated the association of thyroid hormones with BMI and HOMA-IR in euthyroid subjects. They found that fT3 levels were slightly higher in subjects with a BMI ≥25kg/m2.^13^ Owecki et al.^21^ investigated insulin resistance in patients with hypothyroidism caused by thyroidectomy and found no correlation between hypothyroidism insulin resistance. Dimitriadis et al.^22^ investigated insulin action on adipose tissue and muscle in hypothyroid subjects and found resistance to insulin-stimulated glucose uptake in muscle and adipose tissue. Bougle et al.^23^ found an association between increased serum TSH and decreased insulin resistance in obese subjects and concluded that changes in thyroid function may protect against obesity-related metabolic diseases.

We found no effect of TSH levels on insulin resistance and BMI. Our comparison of insulin resistance and BMI in patients with high-normal (2.5–4.2 µIU/mL) and subclinical (4.2-10 µIU/mL) TSH levels revealed no difference between groups. A study conducted in Taiwan found that subclinical hypothyroidism increased the risk of death, and all-cause death was found to increase 1.68-fold after controlling for age, sex, BMI, DM, hypertension, dyslipidemia, smoking, alcohol intake, physical activity, income, and education level. Moreover, the risks of cardiovascular related-events and all-cause death increased in patients with subclinical hypothyroidism compared with euthyroid individuals.^24^

Based on the results of this study, we plan to further investigate the relationship between insulin resistance and BMI in subjects with various TSH levels.

## CONCLUSION

HOMA-IR and BMI are important for the assessment of DM and cardiovascular risk. Several previous studies of the association between thyroid disease and HOMA-IR and BMI levels have yielded controversial findings showing both positive and negative results. Our results are consistent with those studies showing no association between TSH levels and HOMA-IR and BMI.
